# Dopamine in Autism Spectrum Disorders—Focus on D2/D3 Partial Agonists and Their Possible Use in Treatment

**DOI:** 10.3389/fpsyt.2021.787097

**Published:** 2022-02-03

**Authors:** Vanja Mandic-Maravic, Roberto Grujicic, Luka Milutinovic, Ana Munjiza-Jovanovic, Milica Pejovic-Milovancevic

**Affiliations:** ^1^Institute of Mental Health, Belgrade, Serbia; ^2^Faculty of Medicine, University of Belgrade, Belgrade, Serbia

**Keywords:** autism, D2 receptors, D3 receptors, dopamine, autism spectrum disorders

## Abstract

Autism spectrum disorders (ASD) are a group of disorders characterized by impairment in social communication and repetitive and stereotyped behaviors. ASD etiology is very complex, including the effect of both genetic and environmental factors. So far, no specific treatment for the core symptoms of ASD has been developed, although attempts have been made for the treatment of repetitive behavior. The pharmacological treatment is aimed at treating non-specific symptoms such as irritability and aggression. Recent studies pointed out to the possible role of altered dopamine signaling in mesocorticolimbic and nigrostriatal circuits in ASD. In addition, several research pointed out to the association of dopamine receptors polymorphism and ASD, specifically repetitive and stereotyped behavior. In this paper, we will provide a review of the studies regarding dopamine signaling in ASD, existing data on the effects of D2/D3 partial agonists in ASD, possible implications regarding their individual receptor profiles, and future perspectives of their possible use in ASD treatment.

## Introduction

Autism spectrum disorders (ASDs) are a heterogeneous group of disorders with primary characteristics being impairment in social development and communication, associated with the presence of repetitive behaviors and restricted interests (1). ASD have been in the focus of research in the past decades, predominantly due to the rise in their prevalence. Namely, recent studies have shown that about 16.8 per 1,000 (one in 59) children aged 8 years are diagnosed with ASD (2).

Despite the increasing clinical and scientific interest in ASD, it is still a group of disorders defined only by clinical manifestations, without defined etiopathogenetic causes (1, 3).

The recommendation for ASD treatment, especially in children, are educational and behavioral interventions (4). However, recent studies show that 27–50% of persons with ASD are treated with medication (5–7). The prevalence of medication use in ASD rises with age and comorbidities (7). Without a known and well-defined underlying cause, pharmacotherapy of ASD is mostly oriented toward the controlling of associated symptoms of ASD, while there is still no evidence-based pharmacological intervention that can be used for the core symptoms of this group of disorders (8). A well-defined group of maladaptive behaviors, which includes aggression, self-injury, stereotypies. and tantrums, usually requires pharmacotherapy (1). These symptoms are seen in up to 85% of children with ASD (9) and are usually the targeted symptoms in ASD treatment with medication (10, 11).

The mostly used treatment so far was oriented toward using serotonin and dopamine-related medication. For selective serotonin reuptake inhibitor (SSRI) antidepressants, there were some positive, but conflicting results only for fluoxetine and fluvoxamine (studies with adults), while there could not be enough data to support recommendation of SSRI in the treatment of repetitive behavior in children and adults with ASD (12).

When it comes to antipsychotics, however, the benefit is somewhat more documented. The first studies from the 1980's showed benefit from the use of first generation antipsychotics (FGA), such as haloperidol, mostly on decreasing hyperactivity, stereotypic behaviors, aggressiveness, and tantrums, without the beneficial effect on learning (13). A much larger body of evidence can be found regarding the efficacy of risperidone in the treatment of both aggression/impulsivity and stereotyped behavior (14, 15). It was Food and Drug Administration (FDA) approved in 2005, for the treatment of irritability in ASD, including tantrums, aggression, and self-injury (16)—but not for stereotyped behavior.

For olanzapine, there has only been one pilot randomized controlled trial (RCT) in children with ASD (17). In this study, the Clinical Global Impression—Improvement scale (CGI-I) was improved 50% in the olanzapine group vs. 20% in the placebo group; although it showed promising results for the overall functioning in ASD, a significant weight gain remained a limiting factor for its use (17). Quetiapine has not been examined in an RCT so far, but an open-label study showed significant reduction in aggression and improvement of sleep in children and adolescents with ASD (18). No RCTs were done for clozapine and ziprasidone, as well (16), although in open label studies, these medications were found to be effective in treatment of aggression and irritability (16). One study compared the clinical efficacy of amisulpride and bromocriptine in a randomized, double-blind, crossover trial in nine children with ASD. Amisulpride had no effect on the overall autistic behavior but was effective in treating negative symptomatology, such as inhibition and withdrawal (19).

In spite of some positive effects in the reduction in maladaptive behaviors, the adverse effects of the regularly used pharmacotherapy are significant and limit their use in children and adults with ASD. A study done in 202 subjects with ASD showed that treatment with risperidone, aripiprazole, and olanzapine resulted in statistically significant increase in body mass index (BMI) z-score, while this was not the case with ziprasidone and quetiapine. The greatest increase in BMI was shown for olanzapine (20).

We will review the current state regarding the role of D2/D3 partial agonists in the treatment of ASD and further explore the future possibilities of their use in ASD treatment—in general and on specific symptoms of this group of disorders, specifically considering their better adverse effects profile in comparison to other FGA and second generation antipsychotics (SGA). Before that, we will briefly present the dopamine theory of ASD, with a specific focus on D2/D3 receptors.

## The Dopamine Theory of Autism Spectrum Disorders

Although there has been an abundance of research related to the etiology and pathophysiology of ASD in the last two decades, the researchers are still in the dark when it comes to the mechanisms that take part in the pathogenesis of ASD (1).

The two modulatory centers of the brain that mainly modulate core traits of ASD through their rich projections are the ventral tegmental area (VTA) and substantia nigra (SN), respectively (21). The similarities in the clinical presentation of ASD to other psychiatric conditions (e.g., schizophrenia) lead to the hypothesis that the basic pathogenic process is related to the dysfunction of the dopaminergic signaling system in certain brain areas (22).

Dopamine (DA) is indeed one of the main neurotransmitters in charge of social behavior and social cognition and of control of movement (23, 24). Several researchers in this area proposed a framework that clarifies the role of the dopaminergic system in ASD (22, 25, 26).

A network of brain regions (amygdala, ventral striatum, and prefrontal cortex) works in synergy to produce different aspects of social motivation and social behavior (27). The fine alterations in this network correlate with individual differences in social motivation; e.g., anti-social personality traits in certain individuals are associated with the lesser activity in these areas (28). These processes are controlled mainly through the mesocorticolimbic (MCL) pathway, a pathway known to guide reward and motivation-related behavior (29). Research has shown that MCL connections regulate this behavior mainly through the dopaminergic projections from VTA to the nucleus accumbens and the prefrontal cortex. More specifically, the research performed on animal models confirmed that the activation of VTA leads to the activation of D1 receptors, which consequently stimulates the social interaction in animals. In contrast, the inhibition of the same area had the opposite effect—social inhibition (29).

Dichter et al. first summarized all current evidence related to the DA pathway changes in ASD and provided a new perspective in approach to ASD, mainly through the “reward-circuitry” dysfunction (25). More specifically, individuals with ASD have functional alterations in the DA mesocorticolimbic signaling pathway. These alterations include the reduction in DA release in the prefrontal cortical area and diminished responsiveness of nucleus accumbens (30). Additionally, there is evidence that ASD is related to the general hypoactivation of the reward system (31). New genetic research has discovered genetic variants and mutations of dopamine transporter (DAT) that alter dopamine transmission and consequently lead to ASD-like behavior patterns (32, 33). However, as in the majority of neurodevelopmental conditions, the susceptible genotype is not always enough to explain the occurrence of ASD. The interaction between genes and environment has proven to be the model that explains the abnormal dopamine transmission and ASD-like behavior in animal models (34, 35).

According to some authors, the alterations in dopaminergic transmission could be the cause of reduced motivation to pursue social interactions, since the brain of autistic individuals could register these activities as “not rewarding.” The reduced motivation also leads to reduced social experience and consequently to deficits in the development of social cognition.

The other important dopaminergic circuit that supports the dopaminergic theory of ASD is the nigrostriatal circuit (NS). This circuit arises from the neural projections from the substantia nigra toward the dorsal striatum. The NS modulates the motor aspects of goal-directed behavior to produce suitable actions for a specific outcome (27). Considering this crucial role of NS, it is not surprising that the dysfunction of this neural circuit could result in loops of purposeless, stereotyped patterns of behavior typical for ASD. Moreover, animal studies have proven that drug-induced dysfunction of this circuit caused stereotyped autistic-like behavior in mice (26). The treatment of these behaviors using D1/D2 dopaminergic receptor blockers leads to their reduction (36). This finding has opened a new treatment possibility in individuals with ASD.

Recently, immune alterations in ASD have been demonstrated in multiple studies, and a link between this alteration and brain maturation, and dopaminergic pathways is currently being intensively studied. A few molecular signaling pathways have been recognized linking immune activation to ASD phenotypes, including cytokine pathways. It was recently shown that children with ASD had increased interleukin (IL)-31 messenger RNA (mRNA) and protein expression levels, and elevated interleukin 16 expression compared to typically developing children (37, 38). Not only cytokines play an important role, but also CD45 cells have a key role in the pathogenesis of several autoimmune disorders (39). Ahmad and coworkers have shown that children with ASD exhibited significantly higher numbers of CD45^+^GM-CSF^+^, and other proinflammatory mediators such as CD45^+^IFN-γ^+^, CD45^+^IL-6^+^, CD45^+^IL-9^+^, CD45^+^IL-22^+^, CD45^+^T-bet^+^, and CD45^+^pStat3^+^ cells, compared with the control group (40).

On the other hand, research on animal models of ASD also correlated immunological alteration with alteration in transcription factor signaling pathways (41–43). It was shown in animal models that immune activation at late stages of the embryonic brain development initiates the activation and alteration in expression of multiple receptors in different signaling pathways (including immunological) that causes changes in neuronal migration and production of interneurons. For example, maternal immune activation at late gestation day in animal models altered the expression of neuregulin 1 (NRG1), its receptor tyrosine-protein kinase (ErbB4), and NRG1-ErbB4 pathway and also consequently or inherently lead to alteration in dopamine D2 receptor with further resulting in cognitive dysfunction (44). Other data suggest that early prenatal stress induces both alteration in expression of dopamine D1 and D2 receptors and increased levels of immune response genes, including the proinflammatory cytokines IL-6 and IL-1β (45).

As it was shown, several studies pointed out to the possibility of a link between immune alteration, dopaminergic pathways and ASD (44, 45), adding to the complexity and the potential significance of the dopamine theory of ASD.

## The Role of D2 and D3 Receptors and Their Possible Implications in ASD

D2 receptor (D2R) and D3 receptor (D3R) belong to the class-2 dopamine receptors (DRD2, DRD3 and DRD4). Their mechanism of action is manifested *via* inhibition of the cAMP production by coupling to Gi/o G proteins (46).

D2Rs are expressed throughout the brain and are localized both on presynaptic dopaminergic neurons and postsynaptic neurons targeted by dopaminergic afferences (47). That way, D2Rs have a function of modulating the DA release, while as heteroreceptors, they modulate neurotransmitter release from postsynaptic neurons, as well.

D2Rs are densely present in the striatum, while extrastriatal D2R are also detected in the cortex, mostly in the temporal, frontal, occipital, prefrontal, and anterior cingulate cortices (46, 48). The cortical density of D2Rs is 2–8% of the density found in the putamen (49).

Interestingly, D2Rs are expressed mostly in cortical areas involved in the processing of emotional and sensory-motor modalities. Variations in expression of D2Rs in different brain regions might be associated with various symptoms in psychiatric disorders (46).

A recent study was done on postmortem basal ganglia (BG) of persons with ASD comparing to neurotypical controls (50). The basal ganglia (BG) are important in action selection, learned habits, action sequences, and repetitive behaviors (50). The study showed significant elevation in D2Rs mRNA within the medium spiny neurons (MSNs) of the caudate and putamen of persons with ASD, implicating the indirect BG pathway. The indirect BG pathway enables the performing of an action chosen by the direct way, by inhibiting competing motor actions. Therefore, its disturbance might lead to motor dysfunction, stereotypy, and other repetitive behaviors in individuals with ASD (50). Besides this translation to clinical manifestations of ASD, the authors of the study point out to the fact that the BG has also been implicated in the cognitive control of language processing, therefore showing the possible link between DR2s alterations and language impairment in ASD (50).

In addition, it is important to note that D2 receptors in the prefrontal cortex PFC favor fast flexible switching between representations, meaning D2Rs have an important role in cognitive flexibility (51). This function of D2R might, therefore, be impaired in the typical ASD symptom of insistence on sameness.

The localization of D3 receptors is mostly in the limbic system (including nucleus accumbens), which is, as it is already mentioned, important in motivation and reward and also social interaction (52). The important fact is that dopamine D3R are also autoreceptors—presynaptic receptors, with inhibitory effects on dopamine impulse flow, dopamine synthesis, and dopamine release (53). The D3 system is involved in the regulation of cognitive, social, emotional, motivational, and locomotor processes (54).

It was shown that D3 receptors play an important role in cognition and learning (54). The basic rule is that D3R agonism reduces cognition, while D3 antagonism improves cognitive functioning (54). A study by Lemercier et al. showed that the basis of D3R agonism effect lies in the decreased synchronized electrophysiological activities necessary for proper cognitive functioning (55). At the level of neurotransmitters, D3 antagonists promote the release of ACh in the frontal cortex and may potentiate D-serine gating of N-methyl-d-aspartate (NMDA) receptors, making D3 antagonists even a possible choice for treatment of Alzheimer's disease (56).

D3R, more than D2R, are implicated in social interaction and play a significant role in social behavior, with D3R agonists reducing manifestations of social interaction in animals (54, 57). Cariprazine, the D3R partial agonist, has been proven to improve social interactions in animal studies (58).

The role of D3 receptors in locomotor activity has been explored in studies with D3 agonists, showing a biphasic response after their application—hypomotility at low doses and hypermotility at higher doses (54).

Therefore, besides the elements of impaired social functioning, D3 receptor might also be important in terms of repetitive behavior in ASD. The rigid, repetitive actions and stereotypies are affecting individuals with ASD greatly. As already mentioned, these kinds of behavior are often the reason to use pharmacological interventions in children with ASD (59). A study done in 2009 showed that the specific part of repetitive behavior—the insistence on sameness [derived from the Autism Diagnostic Interview (ADI-R)] is associated with polymorphism of the D3 receptor gene (DRD3) in ASD (60). It was also proven to be associated with ASD in a later study (61). These findings are important in terms of possible subphenotyping of persons with ASD. Specific clinical manifestations might have a genetic basis underlying the specific symptoms, not the ASD itself. Besides etiological importance, the subphenotyping might be of great value in terms of pharmacotherapy specifically oriented toward the symptoms (59). If we translate that notion into clinical practice, it might mean that already available drugs acting on D3 receptors might be effective in treating repetitive and stereotyped behavior.

The localization and function of D2 and D3 receptors are presented in Table 1.

**Table 1:** The localization and function of D2 and D3 receptors.

**Type of receptor**	**D_**2**_ Receptors**	**D_**3**_ Receptors**
Localization	Presynaptic and postsynaptic neurones of striatum, cerebral cortex (temporal, prefrontal, frontal, occipital and anterior cingulate cortices), putamen (46, 48)	Presynaptic receptors in limbic system (ventral striatum including nucleus accumbens), thalamus, hippocampus, cerebral cortex, putamen (52, 53)
Mechanism of action	Inhibition in production of cAMP and negative modulation of PKA activity by coupling to Gi/o G proteins and negatively coupling to adenylyl cyclase (AC) (46)	
Function	Aspects of motor function and behavior, language processing, cognition, control of prolactin secretion and alpha MSH secretion from pituitary gland, cardiovascular system function (50, 62).	Aspects of motor function, cognition, emotional processing, social interaction (54, 63)

## The Use of D2/D3 Partial Agonists in ASD

The most important compounds in this group of antipsychotics are aripiprazole, cariprazine, and brexpiprazole (64). D2/D3 partial agonists show different levels of D2 and D3 intrinsic activity, making every compound specific regarding clinical efficacy and safety (65). All of the three compounds have high affinity for the dopamine D2 receptor with brexpiprazole having the highest affinity, followed by aripiprazole and cariprazine (64). D2/D3 partial agonists have intrinsic D2 receptor activity lower than that of dopamine, leading to functional dopamine antagonism (64). Aripiprazole has intrinsic activity of about 20% that of dopamine, while brexpiprazole and cariprazine both have lower intrinsic dopamine activity than aripiprazole, similar to one another [see in (64)].

Studies have shown differences in binding kinetics of aripiprazole and cariprazine (66). Specifically, both aripiprazole and cariprazine show slow dissociation kinetics at the D2 receptor. On the other hand, a significant difference was found regarding D3 receptors. Namely, while aripiprazole shows a slow, monophasic dissociation, cariprazine exhibits a biphasic binding behavior (66). This finding might be translated into cariprazine's *in vivo* action—it might mean that it can react rapidly to variations in the dopamine level (66), which may be important for the reduction of negative symptoms (64). In addition, cariprazine is a D3 preferring D2/D3 partial agonist. This property is unique of cariprazine (67).

In the next section, we will give a short review of the specific D2/D3 partial agonists and their possible use in ASD.

Aripiprazole is an atypical antipsychotic that is FDA approved and predominantly used for management of psychosis in patients with schizophrenia and monotherapy or adjunctive therapy for acute manic episodes associated with bipolar disorder. The oral tablet and solution are also FDA approved for the treatment of ASD. The FDA approved aripiprazole in 2009 for the treatment of irritability in children (ages 6–17 years) with ASD (68). It is considered to be a stabilizer of dopamine and serotonin within the nucleus accumbens, ventral tegmental area, and frontal cortex (69).

A review of three studies suggested that aripiprazole can be effective as a short-term medication intervention for some behavioral aspects of ASD in children/adolescents (70). After a short-term medication intervention with aripiprazole, children/adolescents showed less irritability and hyperactivity and fewer stereotypies. However, notable side effects, such as weight gain, sedation, drooling, and tremor, must be considered. Relapse rates did not differ between children/adolescents randomized to continue aripiprazole vs. children/adolescents randomized to receive placebo, suggesting that re-evaluation of aripiprazole use after a period of stabilization in irritability symptoms is warranted (70).

A 2018 meta-analysis concluded that aripiprazole is efficacious in the acute treatment of irritability, hyperactivity/noncompliance, inappropriate speech, and stereotypic behavior in children and adolescents with ASD (71). On the other hand, it was shown that treatment with aripiprazole did not improve the social withdrawal in such patients. However, it is reasonably safe, more acceptable, and well tolerable in such treatments. In addition to its efficacy in ASD children and adolescents, aripiprazole has shown low risk of adverse events, particularly in cardiovascular, metabolic, and hyperprolactinemic side effects (71). A recent post-marketing surveillance study suggested that aripiprazole was well tolerated and effective in the long-term treatment of irritability associated with ASD in Japanese children and adolescents in the real-world clinical practice (72). Initially, there was an opinion that aripiprazole was safer than risperidone (73). More recent studies stated that there was not much difference in safety and efficacy between the two drugs (74). Another study compared efficacy and tolerability of aripiprazole and risperidone and came to a conclusion that the benefit of aripiprazole treatment seemed significantly greater at 12 weeks but that this difference did not persist at 24 weeks. This could indicate a faster positive effect of aripiprazole compared to risperidone. In this study, aripiprazole and risperidone appeared to have similar benefits in terms of efficacy and tolerability, since both drugs were well tolerated with no serious adverse events detected (75).

Brexpiprazole acts as a partial agonist at 5-HT1A and D2 receptors at similar potencies and as an antagonist at 5-HT2A and adrenergic alpha1B/2C receptors. As it was already mentioned, brexpiprazole has less intrinsic agonist activity at D2 receptor than aripiprazole, suggesting a relatively lower tendency to cause D2 partial agonist-mediated side effects, such as akathisia and restlessness (67). The affinity of brexpiprazole for the 5-HT1A receptor is over 14 times higher than that of aripiprazole and about 22 times higher than that of cariprazine (64). Therefore, the specificity of brexpiprazole might be acting on serotonin 5-HT1A receptor. That way, it might increase dopamine and acetylcholine release in the prefrontal cortex and may be beneficial for improving cognitive dysfunction, negative symptoms, and depression (76). Clinical studies in patients with schizophrenia showed a good profile of adverse effects—the only common adverse event was weight gain (67). Akathisia was not significantly associated with brexpiprazole in comparison to placebo. Most cases were mild or moderate in severity and did not lead to treatment discontinuation (67). There were no head-to-head comparisons with aripiprazole, but given the mechanism of action and recent data, brexpiprazole might be related to less akathisia and more weight gain than the two compounds (67).

To our knowledge, there were no studies of brexpiprazole in ASD, nor in specific age-groups (children or adolescents). A preclinical study showed that brexpiprazole significantly ameliorated dizocilpine-induced social recognition deficits, which was not shown for risperidone or olanzapine in this study. This mechanism might be related to brexpiprazole effect on the 5-HT_1A_ receptor (77). The fact that brexpiprazole might have beneficial effects on social recognition possibly might be explored in future studies in ASD.

When it comes to cariprazine, to our knowledge, there are no studies regarding its efficacy in persons with ASD. Recently, a study on an animal model of ASD was published (60). Namely, a study was done in the rat prenatal valproic acid (VPA) exposure model, and it explored the effects of cariprazine on behavioral endpoints representing the core and associated symptoms of ASD, in comparison to aripiprazole and risperidone (60). Behavioral tests such as employing social play, open field, social approach avoidance, and social recognition memory tests were done in male offspring of rat dams treated with valproates during pregnancy. Cariprazine showed dose-dependent efficacy on all behavioral endpoints and was the only test compound effective in the social play paradigm. In other behavioral measures, cariprazine was equally effective as aripiprazole and risperidone (60). Cariprazine has also been shown to facilitate social interactions in animal models of schizophrenia (58). The beneficial effect on social interactions might be explained by the findings of studies that proved that cariprazine increases dopamine release in the nucleus accumbens and ventral hippocampus (61).

Since this is a finding from an animal study, it is important to understand the implications of the results. The social play has rewarding properties; therefore, dopamine might modulate social play behavior (62). An optimal level of dopamine is required for the expression of social play behavior, while both stimulating and reducing dopaminergic neurotransmission can disrupt social play (62). It was also found that the effect of dopamine on social play is manifested mostly in the nucleus accumbens as the site of action. Blockade of either D1 or D2 NAc dopamine receptors reduced social play in animals highly motivated to play as a result of longer social isolation before testing (52). The authors conclude that the functional activity in the mesolimbic dopamine pathway plays an important role in adaptive social development, whereas abnormal NAc dopamine function may underlie the social impairments observed in developmental psychiatric disorders such as ASD (52).

The specificity of cariprazine's pharmacological profile is its affinity to D3 receptors. In addition, it is important to emphasize that cariprazine's binding affinity is not only higher for the D3 than for the D2 receptor, but also it is even higher than dopamine's affinity for the D3 receptor. That way, with dopamine at physiological doses, cariprazine acts as a D3 receptor blocker, which is not the case with other dopamine partial agonists (63). A PET study done in patients with schizophrenia showed that at dose of 1 mg/day, mean D3R and D2R occupancy was 76 and 45%, respectively, while at the 3 mg/day dose, it was 92 and 79%, respectively (64). These occupancy data first provided evidence that cariprazine is an antipsychotic that dose dependently occupies both the D2R and D3R receptors not only *in vitro* but also *in vivo* with a 3.5–5.5-fold selectivity toward the D3R over the D2R (64).

Taken altogether, with cariprazine expressing high affinity to D3 receptors, it might be a promising medication for intensive repetitive and stereotyped behavior in ASD.

It is hypothesized that cariprazine improves mood, anhedonia in affective disorders, and negative symptoms in schizophrenia specifically with its partial agonist actions at presynaptic D3 autoreceptors in the ventral tegmental area and substantia nigra, causing the disinhibition of dopamine release in the prefrontal cortex, leading to positive dopamine tone (63).

A study done in 2017 by Nemeth et al. compared the effect of cariprazine vs. risperidone in patients with schizophrenia and predominant negative symptoms (78). Patients treated with cariprazine had a greater improvement in predominant negative symptoms of schizophrenia than did patients given risperidone. Additionally, greater improvement for patients given cariprazine vs. risperidone was seen in self-care, personal and social relationships, and socially useful activities (78).

In line with this finding, it is important to note that schizophrenia and ASD share common genetic risk factors and symptom presentations (79, 80) and that there is a significant clinical and biological overlap between the negative symptoms in schizophrenia and ASD. Negative symptoms in schizophrenia include symptoms such as reduced affective sharing and eye contact and lack of social recreational interest, while similarly, one of the core features of ASD includes deficits in social interaction (such as reduced sharing of emotion or lack of social initiation, reduced eye contact, and limited range of facial expressions) (79, 81). Hence, there is a suggested overlap between ASD and schizophrenia, in terms of impairment of social and communicative functioning. The clinical overlap has been suggested in studies showing the same patterns of social cognition between negative schizophrenia and ASD, possibly implying the same neural basis of specific social presentation (79, 82).

Having said that, the documented beneficial effect of cariprazine on negative symptoms in schizophrenia might be translated to the potential beneficial effect of cariprazine on the social impairment of ASD as well.

There are only few studies regarding the tolerability and safety of cariprazine in children and adolescents, in a population with bipolar disorder (65) and schizophrenia (66). In a retrospective study, cariprazine was proven to be well tolerable and effective, but it was done on a small sample (16 patients). There were no serious adverse events, and the main side effect was weight gain. BMI before and after treatment did not change significantly, and weight gain was greater in patients receiving higher doses of cariprazine (≥4.5 mg/day) (65). In another study on 49 adolescents (13–18 years) with the diagnosis of schizophrenia, cariprazine was proven to be well tolerated during the 28-day period. There were no reported changes in the vital signs, laboratory findings, or ECG. Cariprazine did not cause parkinsonism in this study, while akathisia was shown, regardless of the dosing regimen (66).

## Conclusion

As it was shown, there is no pharmacotherapy oriented toward the core symptoms of ASD. Most pharmacological treatment is oriented toward maladaptive behaviors, such as aggression, self-injury, stereotypies, and tantrums. Two core groups of symptoms of ASD—impairment of social interaction and repetitive and stereotyped behaviors—might be, at least partially, explained through the dopamine hypothesis of ASD. When taking into account their localization and function, D2 and D3 receptors might be connected to these symptoms.

The D2R might be connected to stereotypy, and other repetitive behaviors, and language impairment in ASD. This hypothesis might already have a confirmation, since the FDA-approved agents, namely, aripiprazole and risperidone, show action on D2 receptors and improvement in stereotypies in persons with ASD (11, 55).

D3Rs more than D2Rs are implicated in social interaction (57) and cognition and learning (44). Clinically, polymorphism in D3R gene was also connected to insistence on sameness in persons with ASD (60).

Having said that, the group of D2/D3 partial agonists might be a potentially promising therapeutic option, not only for associated but also some of the core symptoms in ASD.

To our knowledge, there are no studies exploring the therapeutic effect of brexpiprazole or cariprazine in persons with ASD.

When everything is taken into account, having in mind the specific receptor profile of cariprazine, and its rather safe adverse events profile, one of the next steps could be directed toward the further exploration of its treatment potential in children and adults with ASD. RCT studies are needed to explore whether the specific pharmacological profile leads to specific clinical changes in this group of disorders.

Future studies might show whether pharmacological agents acting as dopamine “stabilizers” on these receptors have more therapeutic possibilities than those that are currently available and affecting only on non-core symptoms of ASD.

## Author Contributions

VM-M and MP-M designed the study, contributed with their expertise in the process of literature research, and reviewing process. RG, LM, and AM-J contributed with literature research, shaping up the article, and technical work. VM-M, RG, LM, AM-J, and MP-M interpretation and writing the article. All authors contributed to the article and approved the submitted version.

## Conflict of Interest

The authors declare that the research was conducted in the absence of any commercial or financial relationships that could be construed as a potential conflict of interest.

## Publisher's Note

All claims expressed in this article are solely those of the authors and do not necessarily represent those of their affiliated organizations, or those of the publisher, the editors and the reviewers. Any product that may be evaluated in this article, or claim that may be made by its manufacturer, is not guaranteed or endorsed by the publisher.
